# Fast and efficient room-temperature phosphorescence from metal-free organic molecular liquids

**DOI:** 10.1039/d5sc03768a

**Published:** 2025-08-07

**Authors:** Yosuke Tani, Yuya Oshima, Rika Okada, Jun Fujimura, Yuji Miyazaki, Motohiro Nakano, Osamu Urakawa, Tadashi Inoue, Takumi Ehara, Kiyoshi Miyata, Ken Onda, Takuji Ogawa

**Affiliations:** a Department of Chemistry, Graduate School of Science, Osaka University Toyonaka Osaka 560-0043 Japan; b Innovative Catalysis Science Division, Institute for Open and Transdisciplinary Research Initiatives (ICS-OTRI), Osaka University Suita Osaka 565-0871 Japan; c Institute of Transformative Bio-Molecules (ITbM), Nagoya University Furo, Chikusa Nagoya 464-8601 Japan tani.yosuke.y1@f.mail.nagoya-u.ac.jp; d Research Center for Thermal and Entropic Science, Graduate School of Science, Osaka University Toyonaka Osaka 560-0043 Japan; e Department of Macromolecular Science, Graduate School of Science, Osaka University Toyonaka Osaka 560-0043 Japan; f Department of Chemistry, Faculty of Science, Kyushu University 744 Motooka, Nishi Fukuoka 819-0395 Japan

## Abstract

Liquid is the most flexible state of condensed matter and shows promise as a functional soft material. However, these same characteristics make it challenging to achieve efficient room-temperature phosphorescence (RTP) from metal-free organic molecular liquids. Herein, we report efficient RTP from liquefied thienyl diketones bearing one or two dimethyloctylsilyl (DMOS) substituents. These solvent-free liquids exhibit high RTP quantum yields up to 5.6% in air and 25.6% under Ar due to their large RTP rate constant exceeding 5000 s^−1^. Both liquids undergo excited-state conformational changes and afford monomer RTP, exhibiting essentially the same narrowband spectra as in solution. Moreover, introducing two DMOS substituents sufficiently suppresses aggregation-caused quenching of the molecularly emissive phosphors, illustrating a design principle for RTP-active liquid materials.

## Abstract 2

Solvent-free molecular liquids exhibiting highly efficient room-temperature phosphorescence are reported.

## Graphical abstract

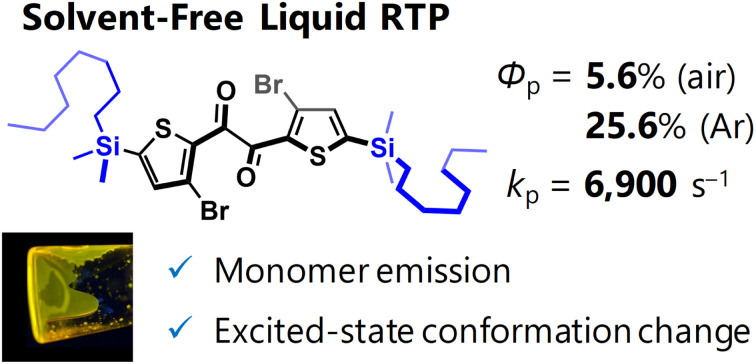

## Introduction

Room-temperature phosphorescence (RTP) from metal-free organic molecules has been actively investigated because of its potential applications in optoelectronics and bioimaging.^1^ Considering an increasing demand for soft materials, developing luminescent solvent-free liquids is of significant interest, since they are easily processed and deformable, while they can accommodate high brightness owing to the high chromophore density.^2^ However, achieving efficient RTP from organic molecular liquids is quite challenging. Established approaches to organic RTP generally require controlled intermolecular interactions and molecular arrangements in rigid crystals or host–guest systems.^3^ This limitation is mainly because the radiative decay (*i.e.*, phosphorescence) of organic compounds is inherently slow due to its spin-forbidden nature. Typical value of phosphorescence rate constant *k*_p_ for metal complexes is 10^4^–10^5^ s^−1^, while that of metal-free organic molecules is 1–100 s^−1^.^4^ As a result, RTP would be easily outpaced by nonradiative decay under non-rigid flexible environments without strong intermolecular interactions. In addition, interchromophoric interactions in the condensed state often cause concentration quenching.^2,4*b*,5^

**Fig. 1:**
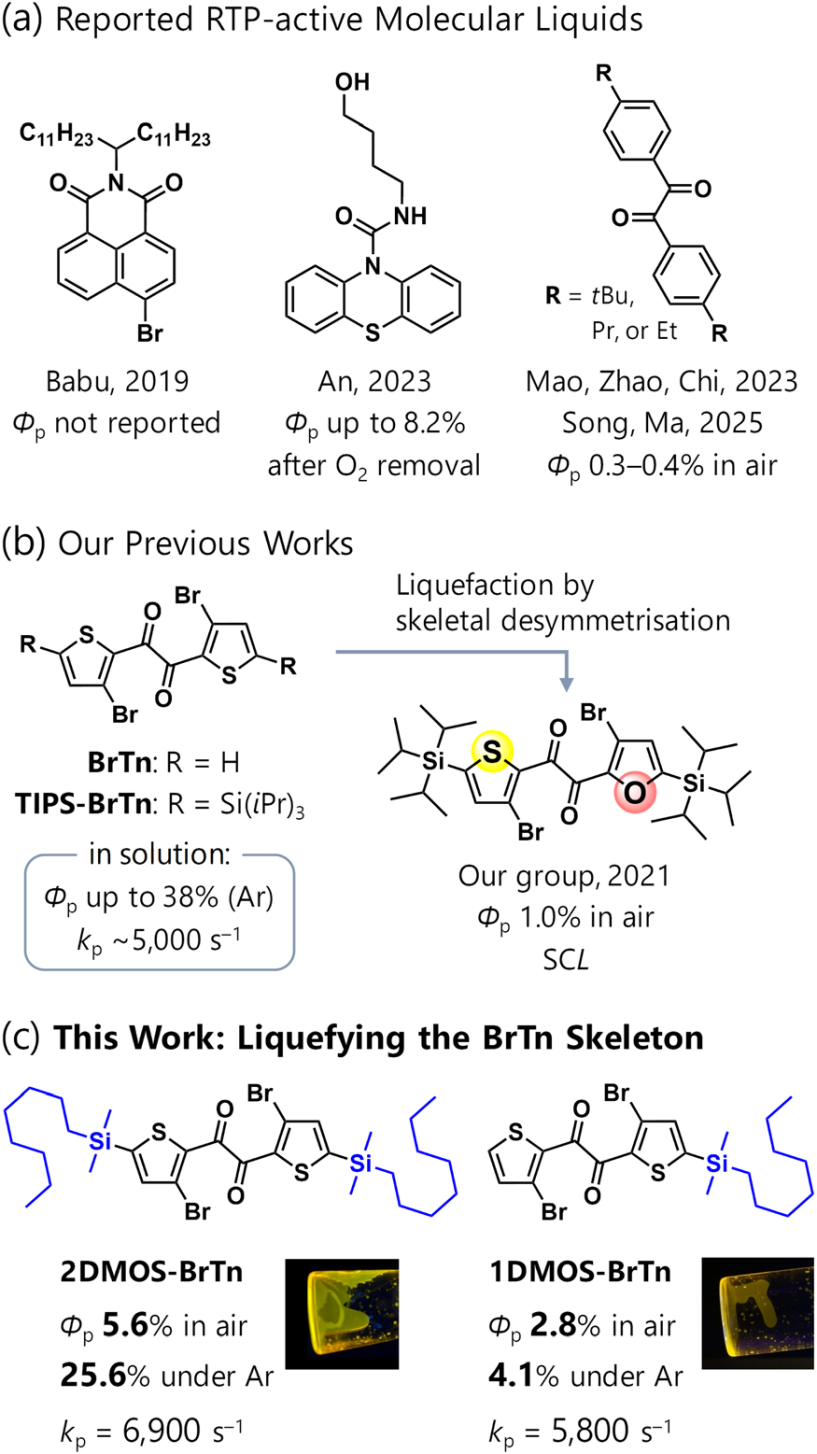
Background of metal-free organic molecular liquids exhibiting room-temperature phosphorescence (RTP). *Φ*_p_, RTP quantum yields: *k*_p_, phosphorescence rate constant. These values are of its solvent-free liquid state unless otherwise noted. SCL, supercooled liquid.

Despite these difficulties, there are a few encouraging precedents on RTP from organic solvent-free liquids (Fig. 1a). In 2019, Babu *et al.* reported a pioneering work on an organic RTP from an alkylated bromonaphthalimide in its solvent-free liquid state.^6^ Although the steady-state total photoluminescence (PL) was dominated by fluorescence with a quantum yield of 0.1%, indicating the low RTP efficiency, a long lifetime of 5.7 ms was achieved. In 2023, An *et al.* reported an efficient organic RTP from a supercooled liquid (SCL) state, *i.e.*, a kinetically-trapped metastable liquid state below its melting point, of a 10*H*-phenothiazine-10-carboxyamide bearing a hydroxy-terminated alkyl chain.^7^ Its intermolecular hydrogen-bonding suppresses the nonradiative decay, and the RTP efficiency *Φ*_p_ is reported to reach 8.2% after oxygen removal by photoirradiation. In the same year, Mao, Zhao, and Chi *et al.* reported RTP from a melt-quenched SCL state of 4,4′-*tert*-butylbenzil with *Φ*_p_ = 0.33% in air.^8^ During the review of the present paper, Song and Ma *et al.* reported that two other benzils with ethyl or propyl groups instead of *tert*-butyl groups also show RTP in the liquid state with *Φ*_p_ = 0.4 and 0.3% in air.^9^ Notably, all these works liquefied chromophores whose molecular RTP efficiency (*e.g.* that in solution) was very low. As a result, only An's example, which was designed to suppress the nonradiative decay by strong intermolecular interactions, exhibited *Φ*_p_ over 1%.

Recently, we reported a highly efficient RTP from brominated thienyl diketone (bromo-thenil, BrTn) derivatives in solution, with *Φ*_p_ up to 38% under Ar (Fig. 1b left).^10^ Detailed investigations revealed that the efficient RTP in solution stems from the significant *k*_p_ of ∼5000 s^−1^, which is close to that of Pt porphyrin complexes. We envisioned that the fast nature of the RTP would be promising for realising efficient RTP from a molecular liquid. However, BrTn exhibits high crystallinity (melting point: *ca.* 180 °C) and suffers from aggregation-caused quenching (ACQ), showing no emission in the crystalline state.^11^ Therefore, molecular design of liquefaction while avoiding ACQ of RTP is required. In 2021, we developed an RTP-emitting SCL by desymmetrising the BrTn skeleton bearing bulky tri(isopropylsilyl) (TIPS) substituents (Fig. 1b right).^12^ The resulting liquid thienyl furyl diketone exhibits RTP-dominated emission with a relatively high *Φ*_p_ of 1.0% in air, and the unsymmetrical structure provided high kinetic stability of the SCL state.^13^ Importantly, SCL is a metastable phase. SCLs change their properties drastically upon isothermal liquid–solid phase transition, which can be utilised to develop stimulus-responsive materials.^14^ On the other hand, SCLs may undergo unexpected crystallisation during processing, device fabrication, and long-term use and storage. In this aspect, stable liquids are more desirable. Moreover, the *k*_p_ value of the liquid thienyl furyl diketone was estimated to be 650 s^−1^. This value is quite high for a metal-free organic molecule, whereas that of BrTn is even higher.

Herein, we liquefied BrTn phosphor by introducing two or one dimethyloctylsilyl (DMOS) groups. As a result, stable liquids with reasonable viscosity were obtained. They exhibit efficient RTP with *Φ*_p_ up to 5.6% in air and 25.6% under Ar, attributed to the significant *k*_p_ values (Fig. 1c). The introduction of two DMOS substituents effectively suppressed ACQ, providing a slightly higher *Φ*_p_ than in solution. Moreover, the UV-visible absorption and photoluminescence (PL) spectra of the solvent-free liquids closely matched their solution-state counterparts. This spectral correspondence is an indicative of monomer RTP behaviour, *i.e.*, the absence of excimer formation and intermolecular electronic or excitonic couplings. These results establish a design approach for developing RTP-active liquids based on molecular phosphorescence.

## Results and discussion

### Synthesis and physical properties of the liquid diketones

The diketones 2DMOS-BrTn and 1DMOS-BrTn were prepared by homo- or cross-benzoin condensation of the corresponding aldehydes, followed by oxidation. After purification using silica-gel column and recycling gel-permeation chromatography (GPC), overnight solvent removal *in vacuo* yielded orange liquids. As-obtained diketones were pure and solvent-free based on ^1^H and ^13^C NMR spectroscopy, elemental analysis, and recycling GPC (see SI for details).

**Fig. 2:**
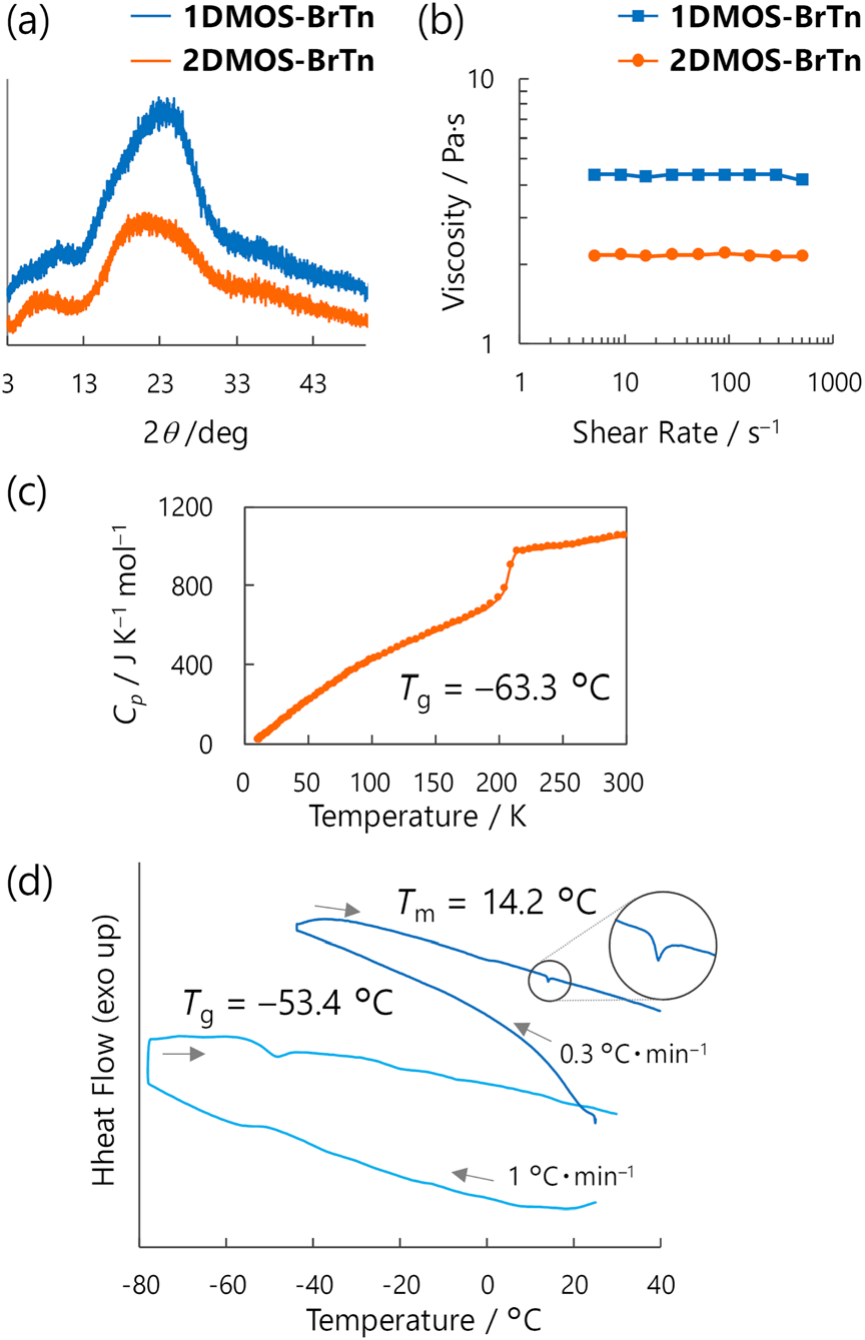
(a) X-ray diffraction profiles of diketones. (b) Steady-flow viscosity of diketones as a function of shear rate. (c) Temperature dependence of heat capacity of 2DMOS-BrTn. (d) Temperature dependence of differential scanning calorimetry of 1DMOS-BrTn.

X-ray diffractometry only exhibited a broad halo, indicating the isotropic nature of the liquids (Fig. 2a). The approximate maxima of the halo appeared at 21° (2DMOS-BrTn) and 24° (1DMOS-BrTn), which correspond to 4.2 and 3.7 Å, respectively, and are assignable to an average distance between the alkyl chains.^2^ Steady-flow viscosity at 25 °C was determined to be 2.1 and 4.4 Pa s for 2DMOS-BrTn and 1DMOS-BrTn, respectively (Fig. 2b), which are within a typical viscosity value for organic molecular liquids.^5,15^ These results imply a denser environment for 1DMOS-BrTn.

Thermal analyses revealed that both diketones are (practically) stable liquids at room temperature (Fig. 2c and d). 2DMOS-BrTn exhibited glass transition (*T*_g_ = −63.3 °C) and did not crystallise during adiabatic heat capacity measurements using a laboratory-made adiabatic calorimeter in the temperature range 10–300 K (Fig. 2c).^16^ The phase behaviour of 1DMOS-BrTn was examined by conventional differential scanning calorimetry (DSC). In the heating trace at 1 °C min^−1^, 1DMOS-BrTn exhibited a broad *T*_g_ at −53 °C and no melting peak (Fig. 2d). According to Nakanishi and co-workers' work on a practical technique to evaluate phase behaviour using DSC,^17^ we cooled down 1DMOS-BrTn to slightly above its *T*_g_ and heated up at 0.3 °C min^−1^; it crystallised around 0 °C and a melting peak appeared at *T*_m_ = 14.2 °C, which is below room temperature. Thus, 1DMOS-BrTn was confirmed to be a thermodynamically stable liquid at room temperature. Below 14.2 °C, 1DMOS-BrTn was in an SCL state with a high kinetic stability, as no crystallisation was observed during storage in a refrigerator for more than two years.

The introduction of only one DMOS substituent dramatically decreased the *T*_m_ from 179.0 °C (BrTn) to 14.2 °C (1DMOS-BrTn). In contrast, *T*_m_ of TIPS-BrTn bearing two TIPS groups was 161.8 °C, which is comparable to that of BrTn.^11,18^ TIPS group is more rigid and sterically demanding than DMOS group, and would reduce interactions between central aromatic moieties. However, TIPS groups had a limited effect on *T*_m_, while flexible DMOS groups had a more significant effect. These observations imply an entropy-driven liquefaction in the 2DMOS-BrTn and 1DMOS-BrTn.

### Photophysical properties in solutions

**Fig. 3:**
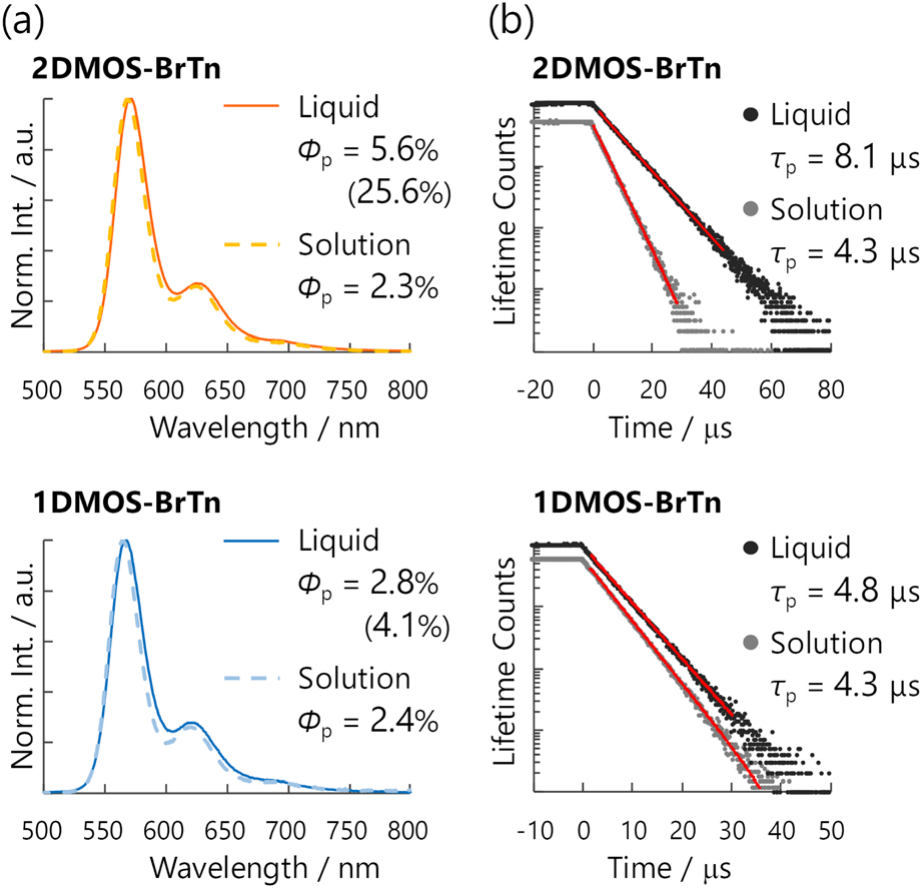
Steady-state photoluminescence (PL) spectra (a) and PL decay curves (b) of 2DMOS-BrTn (top) and 1DMOS-BrTn (bottom) in solution (1.0 × 10^−5^ M in cyclohexane) and solvent-free liquid state, evaluated at room temperature in air. Red lines in panel b denote the fit to the curve. *Φ*_p_, RTP quantum yields; *τ*_p_, phosphorescence lifetimes. Values in parentheses are *Φ*_p_ evaluated under Ar.

To clarify the molecular RTP characteristics, the photophysical properties of the newly synthesised diketones were evaluated in a cyclohexane solution (1.0 × 10^−5^ M) at room temperature in air. The steady-state PL spectra were almost identical to the RTP from BrTn, exhibiting a sharp emission peak at around 570 nm, accompanied by weak vibronic bands (Fig. 3a). The full-widths at half-maxima (FWHM) of the spectra were only 31 and 30 nm for 2DMOS-BrTn and 1DMOS-BrTn, respectively, representing the narrowband emission with high colour purity.^10,19^ In addition, their emission lifetimes were on the order of microseconds (4.3 μs) in air for both (Fig. 3b). The lifetime and the intensity of the emission increased under Ar, while the spectral shapes remained unchanged (Fig. S1). These results confirmed that RTP dominated the steady-state emissions of 2DMOS-BrTn and 1DMOS-BrTn in cyclohexane, and no fluorescence component was discernible. Their *Φ*_p_ values were 2.3% and 2.4% in air, which were comparable to those for BrTn and TIPS-BrTn.

We further evaluated the fast RTP property based on the kinetic rate constants. The experimentally determined *Φ*_p_ and *τ*_p_ correlate the kinetic rate constants according to the formulas: *Φ*_p_ = *ϕ*_ISC_·*k*_p_/(*k*_p_ + *k*_nr_ + *k*_q_[O_2_]) = *ϕ*_ISC_·*k*_p_·*τ*_p_, where *k*_q_[O_2_] and *k*_nr_ are the rate constants for the oxygen quenching and all other nonradiative decays from the T_1_ state, respectively; *ϕ*_ISC_ is the quantum yield of intersystem crossing (ISC) from the S_1_ to T_1_ state.^4*b*,20^ The formula can be rewritten as *k*_p_ = (1/*ϕ*_ISC_)·*Φ*_p_/*τ*_p_. We previously revealed that the thienyl diketones BrTn and TIPS-BrTn exhibited ultrafast ISC with time constants <10 ps, hence the unity *ϕ*_ISC_, regardless of the TIPS moieties.^10^ Therefore, we assumed the *ϕ*_ISC_ of 2DMOS-BrTn and 1DMOS-BrTn as unity as well. The absence of discernible fluorescence components in the steady-state PL spectra also supports this assumption. Consequently, we derived the *k*_p_ values for 2DMOS-BrTn and 1DMOS-BrTn to be 5300 and 5500 s^−1^, respectively (Table 1). These values are exceptionally large as metal-free organic molecules and are comparable to those of BrTn and TIPS-BrTn (5300 and 5000 s^−1^). Thus, the DMOS substituents had a minor effect on the *k*_p_ values, achieving fast RTP in solution.

**Table 1:** Photophysical properties of 2DMOS-BrTn and 1DMOS-BrTn in the solvent-free liquid state and in cyclohexane solution at room temperature

			*Φ*_p_/%	*τ*_p_/μs	*k*_p_/s^−1^ a	*k*_nr_ + *k*_q_[O_2_]/s^−1^ a	*k*_q_[O_2_]/s^−1^ b
2DMOS-BrTn	Liquid	Air	5.6	8.1	6900	1.2 × 10^5^	1.0 × 10^5^
		Ar	25.6	36.5	7000	2.0 × 10^4^	
	Solution	Air	2.3	4.3	5300	2.3 × 10^5^	2.12 × 10^5^
		Ar	22.5	42.7	5300	1.8 × 10^4^	
1DMOS-BrTn	Liquid	Air	2.8	4.8	5800	2.0 × 10^5^	0.6 × 10^5^
		Ar	4.1	6.8	6000	1.4 × 10^5^	
	Solution	Air	2.4	4.3	5500	2.3 × 10^5^	2.17 × 10^5^
		Ar	30.3	52.1	5800	1.3 × 10^4^	

a Calculated according to the formulas: *k*_p_ = *Φ*_p_/*τ*_p_ and *k*_nr_ + *k*_q_[O_2_] = (1 − *Φ*_p_)/*τ*_p_, assuming unity intersystem crossing efficiency.

b Estimated value by subtracting *k*_nr_ + *k*_q_[O_2_] under Ar from that in air.

### Photophysical properties of the solvent-free liquids

The diketones 2DMOS-BrTn and 1DMOS-BrTn exhibit monomer RTP in the solvent-free liquid state in air; the steady-state PL spectra are almost identical to those in solution (Fig. 3a). Their FWHMs are kept as narrow as 32 nm for both. The PL intensity decayed monoexponentially with *τ*_p_ of 8.1 and 4.8 μs in air for 2DMOS-BrTn and 1DMOS-BrTn, respectively, without any short-lived components (Fig. 3b). Such decay profiles, as well as the identical shape of the steady-state PL spectra under Ar (Fig. S2), indicate the RTP-dominated emission without discernible fluorescence.

**Fig. 4:**
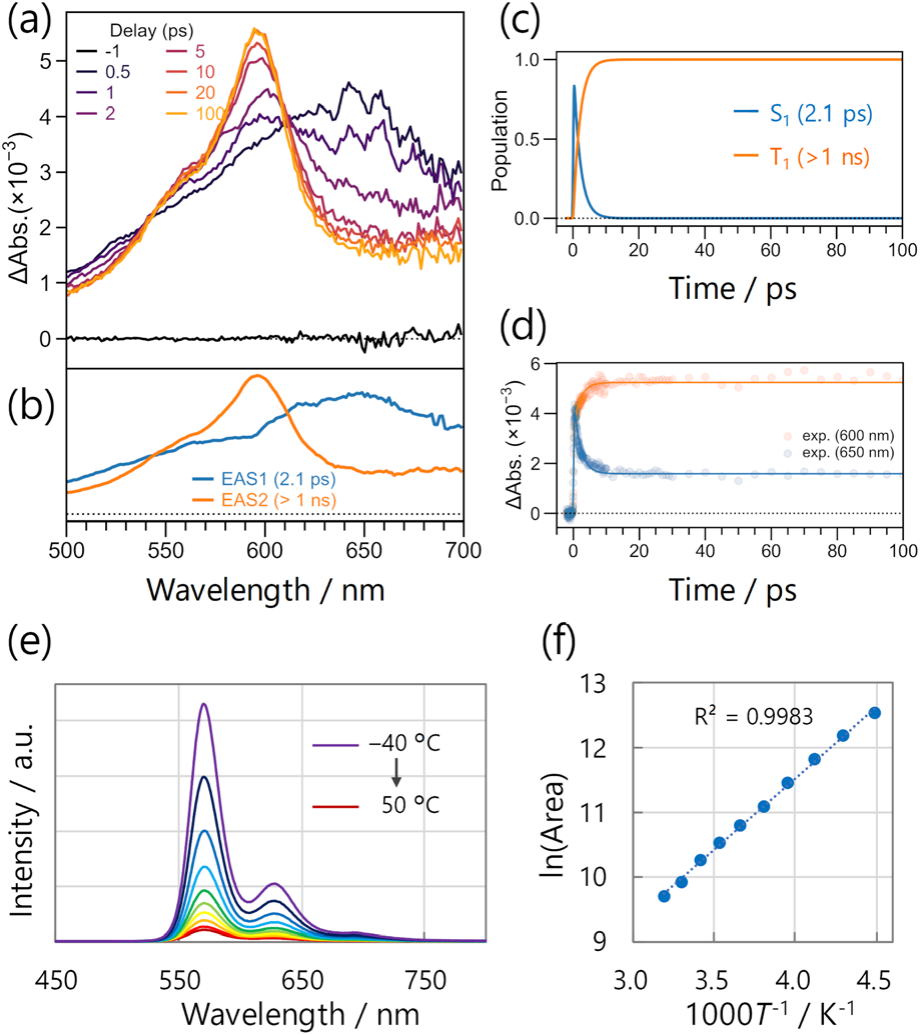
(a) Femtosecond transient absorption spectra of 2DMOS-BrTn in the solvent-free liquid state (excited at 320 nm). (b and c) Selected results from the global analysis based on a sequential model with two states; (b) evolution-associated spectra (EAS) and (c) corresponding concentration kinetics. (d) Fit traces at 600 and 650 nm. (e) Temperature-dependent steady-state PL spectra of solvent-free liquid 2DMOS-BrTn in air (excited at 320 nm). (f) The natural logarithm of the PL spectral area plotted against reciprocal temperature. The dotted line represents a linear fit to the data.

To further clarify the nature of the emission, we measured femtosecond transient absorption (fsTA) on 2DMOS-BrTn in the solvent-free liquid state (Fig. 4a). The TA spectra converged to the excited-state absorption with a peak at 595 nm, which resembles the TA of the T_1_ excited states in thienyl diketones.^10^ This indicates that the T_1_ state is also generated in 2DMOS-BrTn. We analysed the data by global analysis assuming sequential model with two states (Fig. 4b–d). Notably, the T_1_ state was generated with a time constant of 2.1 ps, indicating ultrafast intersystem crossing that outpaces other relaxation pathways. This aligns well with the behaviour of BrTn and TIPS-BrTn in solution; the time constant for generating the T_1_ state was 2.3 ps for TIPS-BrTn. In addition, the temperature dependence of the steady-state PL spectra for the solvent-free liquid 2DMOS-BrTn was examined over a temperature range from −50 to 40 °C. As a result, the PL intensity (area) increased at lower temperatures in a good Arrhenius-type relationship, without emergence of new peaks (Fig. 4e, f, and S4). These findings strongly support the phosphorescence nature of the emission.

Notably, the RTP quantum yields *Φ*_p_ in air were 5.6% and 2.8% for liquids 2DMOS-BrTn and 1DMOS-BrTn, respectively (Fig. 3a and Table 1). These values are, to our knowledge, the highest for the organic RTP of a molecular liquid in the air-saturated condition.^6–8,12^ Moreover, while *Φ*_p_ of liquid 1DMOS-BrTn increased only marginally under Ar from 2.8 to 4.1%, liquid 2DMOS-BrTn demonstrated a remarkable increase of *Φ*_p_ from 5.6 to 25.6%. Such an efficient and narrowband RTP liquid represents a significant potential in applications such as bendable liquid OLEDs.^21^

The primary origin of the efficient RTP from the molecular liquids is their large *k*_p_. We estimated the *k*_p_ values for liquids 2DMOS-BrTn and 1DMOS-BrTn to be 6900 and 5800 s^−1^, respectively, assuming unity *ϕ*_ISC_ on the basis of the ultrafast ISC (Table 1). The *k*_p_ value for liquids 2DMOS-BrTn is 1.2 times larger than that for 1DMOS-BrTn, which could be attributed to difference in external heavy atom effect. Importantly, both of these *k*_p_ values were significantly higher than those for reported liquid organic RTP emitters. However, as the *k*_p_ values are virtually unchanged under Ar, the striking difference in their *Φ*_p_ (25.6% *vs.* 4.1%) and *τ*_p_ (36.5 μs *vs.* 6.8 μs; Fig. S3) under Ar suggests the presence of another critical factor in obtaining efficient RTP, which will be addressed in the following section.

### Contrasting substituent effect in solution and in the solvent-free liquids

Interestingly, the number of silicon substituents has an opposite effect on their nonradiative decay behaviour in solution and in the solvent-free liquid state. In solution, *Φ*_p_ and *τ*_p_ of the two diketones were similar in air; meanwhile, under Ar, *Φ*_p_ and *τ*_p_ of 2DMOS-BrTn (22.5% and 42.7 μs) were smaller and shorter than those of 1DMOS-BrTn (30.3% and 52.1 μs) (Table 1). According to the formula *k*_nr_ + *k*_q_[O_2_] = (1/*ϕ*_ISC_)·(1–*Φ*_p_)/*τ*_p_ and assuming *k*_nr_ >> *k*_q_[O_2_] under Ar, *k*_nr_ of 2DMOS-BrTn is estimated to be 1.8 × 10^4^ s^−1^, which is 1.4 times larger than that of 1DMOS-BrTn (1.3 × 10^4^ s^−1^). Thus, in solution, the DMOS substituent likely induces molecular motions that accelerate nonradiative decay. We previously observed a similar trend in comparing TIPS-BrTn with BrTn.^10^

In contrast, in the solvent-free liquid state, the *k*_nr_ of 2DMOS-BrTn is revealed to be much smaller than that of 1DMOS-BrTn. Thus, *k*_nr_ (∼*k*_nr_ + *k*_q_[O_2_] under Ar) for 2DMOS-BrTn (2.0 × 10^4^ s^−1^) was only one-seventh of that for 1DMOS-BrTn (1.4 × 10^5^ s^−1^; Table 1). Meanwhile, *k*_q_[O_2_] in air, which was estimated by subtracting *k*_nr_ + *k*_q_[O_2_] under Ar from that in air, was larger for 2DMOS-BrTn (1.0 × 10^5^ s^−1^) than for 1DMOS-BrTn (6.0 × 10^4^ s^−1^). These analyses on the liquid-state photophysical properties suggest the following four points. (1) The nonradiative decay pathways other than oxygen quenching (*i.e.*, *k*_nr_) are well suppressed in 2DMOS-BrTn, while they are so fast that they can compete with oxygen quenching in 1DMOS-BrTn. (2) The competing nonradiative decay would be related to intermolecular processes promoted in the condensed state, *i.e.*, processes causing ACQ or concentration quenching. (3) Such decay pathways are suppressed in 2DMOS-BrTn because it is more protected by DMOS substituents, is less dense, and thus has a lower probability to interact with other molecules. (4) Oxygen quenching in liquid 1DMOS-BrTn is slower than 2DMOS-BrTn as a result of the denser and viscous nature of the liquid state, which suppresses the oxygen diffusion rate. It is worth mentioning that the *k*_nr_ value of liquid 2DMOS-BrTn is almost the same as that in solution, indicating the avoidance of ACQ by the two DMOS groups.

### Conformations in the solvent-free liquid state

**Fig. 5:**
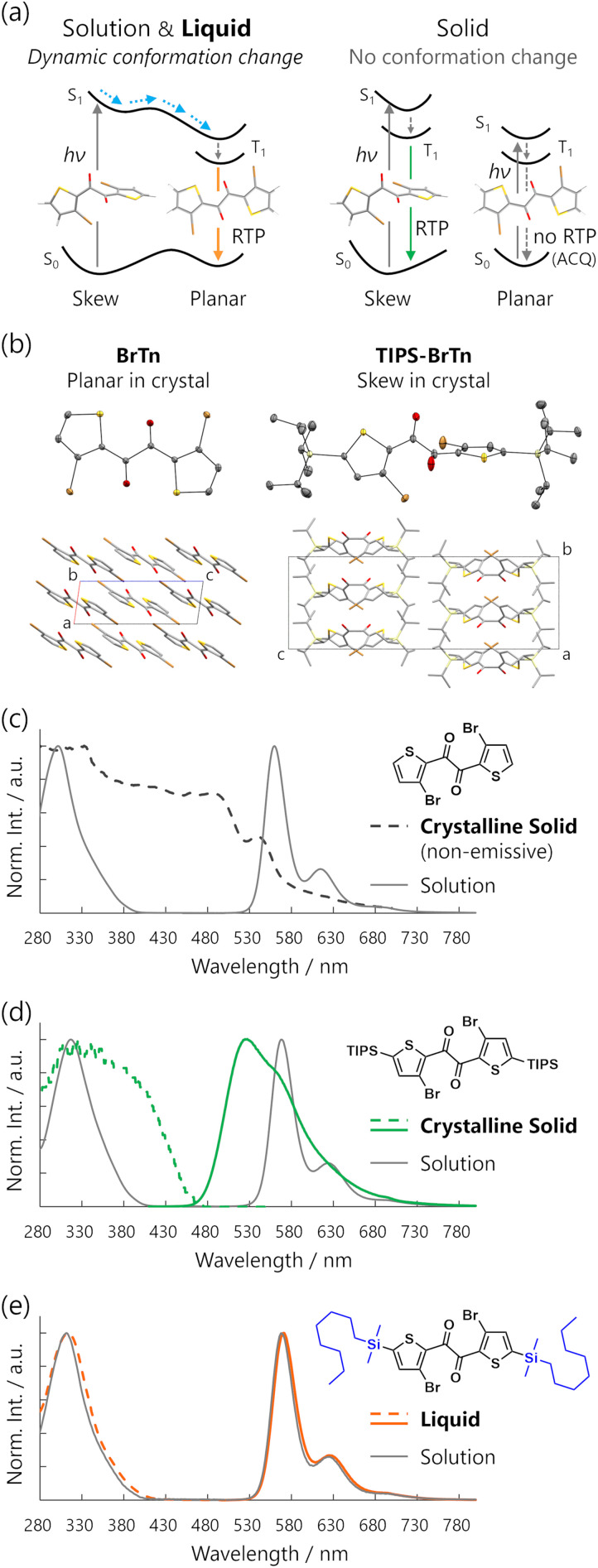
(a) Schematic representation of photophysical pathways of BrTn derivatives in solution and liquid (left) and in solid (right). (b) X-ray crystal structures of BrTn and TIPS-BrTn. Hydrogen atoms were omitted for clarity. (c) Kubelka–Munk converted diffuse reflectance spectrum of solid BrTn (black broken line). (d) Kubelka–Munk converted diffuse reflectance and PL spectra of solid TIPS-BrTn (green lines). (e) Absorption and PL spectra of liquid 2DMOS-BrTn (orange lines). Corresponding absorption and PL spectra in cyclohexane (grey lines, 1.0 × 10^−5^ M) were also shown in panels (c)–(e).

Based on our previous report, BrTn skeleton has two distinct conformers: skew and planar regarding the orientation of the central dicarbonyl moiety (Fig. 5a, left).^10,11,22^ The skew conformer has a shorter π-conjugation length and is the most stable conformer in the ground state, providing absorption maxima at 301 and 317 nm for BrTn and TIPS-BrTn in cyclohexane, respectively (Fig. 5c and d, grey lines). In contrast, the planar conformer has a more extended π-system and is the most stable conformer in the excited state, serving as a source of RTP in solution. In the crystal, TIPS-BrTn exhibits the skew conformation, while unsubstituted BrTn takes the planar conformation, likely due to intermolecular π–π interactions (Fig. 5b).^11^ Thus, the ground-state conformation depends on the intermolecular interactions in the condensed state. Moreover, BrTn exhibits bright RTP in solution while it is nonemissive in the crystal, indicating that interchromophoric interactions of the BrTn skeleton can result in ACQ (Fig. 5a, right; 5c, black broken line). On the other hand, the crystalline solid of TIPS-BrTn exhibits RTP at a shorter wavelength than that of the planar conformer. Therefore, the conformational change is suppressed in the crystal, and the RTP originates from the skew conformer (Fig. 5a, right; 5d, green lines).

The UV-visible absorption spectra of 2DMOS-BrTn and 1DMOS-BrTn in solution are similar to those of BrTn and TIPS-BrTn, with the absorption maxima at 311 and 307 nm, respectively (Fig. 5e, grey lines; Fig. S5). Therefore, they mostly exist as the skew conformer in the ground state in solution. In the solvent-free liquid state, their absorption maxima remained unchanged (Fig. 5e, orange lines; Fig. S5). Thus, 2DMOS-BrTn and 1DMOS-BrTn mainly exist as the skew conformer as well in the solvent-free liquid state without substantial intermolecular electronic interactions. In contrast, the large *k*_p_ and the sharp PL spectral shape observed for liquid 2DMOS-BrTn and 1DMOS- BrTn are the characteristics of the RTP from the planar conformer.^10^ The fsTA spectra also support the assignment of RTP from the planar conformer, as the evolution-associated spectrum-2 corresponds to the T_1_-state planar conformer of the thienyl diketones (Fig. 4b).^10^ These assignments suggest the involvement of the skew-to-planar conformation change in the excited state, which is basically difficult in solid states, resulting in a large gap between the absorption and emission maxima of over 250 nm (Fig. 5a, left; Fig. 5e, orange lines).

Importantly, the excitation spectra matched the absorption spectra (Fig. S5), confirming that the excitation of the skew conformer efficiently generates the excited planar conformer. To assess the possible skew-to-planar intermolecular energy transfer, we evaluated the absorption, PL, and excitation spectra of their dilute solutions in silicone oil (KF-96-3,000CS), whose viscosity (2.9 Pa s) is comparable to the solvent-free liquids 2DMOS-BrTn and 1DMOS-BrTn. As a result, the excitation spectra again matched the absorption spectra (Fig. S6). Therefore, 2DMOS-BrTn and 1DMOS-BrTn undergo skew-to-planar conformation change in the excited state before emitting the RTP, even in the highly viscous solvent-free liquid state.

## Conclusions

Efficient RTP from molecular solvent-free liquid was achieved by liquefying the metal-free organic 3-bromo-2-thienyl diketone skeleton through the introduction of one (1DMOS-BrTn) or two DMOS substituents (2DMOS-BrTn). The RTP quantum yield *Φ*_p_ of 2DMOS-BrTn was 5.6% in air, which, to our knowledge, is the highest value for organic RTP of an air-saturated molecular liquid. Moreover, its *Φ*_p_ was increased to 25.6% under Ar. Notably, although the luminescence of condensed materials often broadens to lose colour purity and suffers from aggregation-caused quenching, the RTP of the liquid diketones were monomer emission, kept the vivid yellow colour, and had a molecular origin. In particular, two DMOS substituents of 2DMOS-BrTn efficiently avoid ACQ, providing slightly higher *Φ*_p_ in the solvent-free liquid state than in solution, both in air and under Ar. Most importantly, the efficient RTP stems from the large *k*_p_ value of over 5000 s^−1^ from the planar conformer. Although the major conformation in the ground state is the skew conformer, it can undergo a conformational change in the excited state, resulting in a large absorption–emission gap of over 250 nm as well as the fast RTP. Such dynamic behaviour represents the uniqueness of the liquid state, the most flexible of condensed matter.

## Author contributions

Y. T. conceptualisation: lead; funding acquisition: lead; investigation: supporting; supervision: lead; visualisation: lead; writing – original draft: lead; writing – review & editing: lead. Y. O. investigation: equal. R. O. investigation: equal. J. F. investigation: supporting. Y. M. investigation: supporting; writing – review & editing: supporting. M. N. investigation: supporting; resources: supporting; writing – review & editing: supporting. O. U. investigation: supporting; writing – review & editing: supporting. T. I. investigation: supporting; resources: supporting; writing – review & editing: supporting. T. E. investigation: supporting; writing – review & editing: supporting. K. M. and K. O. investigation: supporting; resources: supporting; writing – review & editing: supporting. T. O. resources: lead; funding acquisition: supporting; supervision: supporting; writing – review & editing: supporting.

## Conflicts of interest

There are no conflicts to declare.

## Supplementary Material

SC-OLF-D5SC03768A-s001

## Data Availability

The data supporting this article have been included as part of the Supplementary Information. Crystallographic data for BrTn (CCDC 1906439) and TIPS-BrTn (CCDC 1906440) has been deposited at the CCDC and can be obtained from www.ccdc.cam.ac.uk/data_request/cif. CCDC 1906439 and 1906440 contain the supplementary crystallographic data for this paper.^23,24^ Synthesis and characterisation of new compounds and physicochemical properties. See DOI: https://doi.org/10.1039/d5sc03768a.
