# Supplementary material for: Fast and efficient room-temperature phosphorescence from metal-free organic molecular liquids
Source: Chem Sci. 2025 Aug 7;16(37):17480–6. doi: 10.1039/d5sc03768a (PMC12406043; doi:10.1039/d5sc03768a)
Supplement: SC-016-D5SC03768A-s001 [file SC-016-D5SC03768A-s001.pdf]

Supplementary Information (SI) for Chemical Science.
This journal is © The Royal Society of Chemistry 2025

Electronic Supplementary Information

for

Fast and efficient room-temperature phosphorescence from metal-free organic molecular liquids

Yosuke Tani,* Yuya Oshima, Rika Okada, Jun Fujimura, Yuji Miyazaki, Motohiro Nakano, Osamu Urakawa, Tadashi Inoue, Takumi Ehara, Kiyoshi Miyata, Ken Onda, and Takuji Ogawa

Department of Chemistry, Graduate School of Science, Osaka University
1-1 Machikaneyama, Toyonaka, Osaka 560-0043, Japan
Institute of Transformative Bio-Molecules (ITbM), Nagoya University
Furo, Chikusa, Nagoya 464-8601, Japan
E-mail: tani.yosuke.y1@f.mail.nagoya-u.ac.jp

Table of Contents

1. Instrumentation and Chemicals 2
2. Synthesis Procedures 4
3. Additional Photophysical Data 8
4. NMR Spectra 12
5. References 17

1. Instrumentation and Chemicals

Unless otherwise noted, all the reactions were performed under an argon atmosphere using anhydrous solvents and heat-gun-dried glassware on a dual-manifold Schlenk line. ^{1}H and ^{13}C{^{1}H} NMR spectra were recorded on a JEOL ECS400 spectrometer. Chemical shift values (δ) are reported in ppm and are calibrated to tetramethylsilane (0.00 ppm) for ^{1}H and to $CDCl_3$ (77.0 ppm) for ^{13}C NMR. Elemental analysis (EA) was conducted on a Yanaco MT-5 or MT-6 recorder. High-resolution mass spectra (HRMS) were obtained with a Thermo Fisher Scientific Exactive Plus mass spectrometer (ESI-orbitrap) or a Bruker microTOF Focus spectrometry system (APCI). Powder X-ray diffraction (PXRD) patterns were collected on a Rigaku MiniFlex600 with CuKα radiation (λ = 1.5418 Å) using D/teX Ultra as a detector. Rheological measurements were performed at 25 °C using an MCR302 (Anton Paar) rheometer. The liquid sample was placed between the stage and a parallel plate (diameter = 12 mm) of the rheometer and jigged. Differential scanning calorimetry (DSC) was performed with a Hitachi NEXTA DSC200 under a flow of nitrogen.

Analytical thin-layer chromatography (TLC) was performed on aluminum plates bearing a layer of Merck silica gel 60 F_{254}. Column chromatography was carried out on silica-gel 60 (Kanto Chemical Co., Inc., spherical, 63–210 μm). Gel permeation chromatography (GPC) was performed using a JAI LC-9130 NEXT equipped with JAIGEL-1HR and 2HR (eluent: $CHCl_3$, flow rate: 10 mL/min).

Anhydrous THF was purchased from Wako Chemical Co., Inc., and further purified by passage through activated alumina under positive nitrogen pressure as described by Grubbs et al.[1] 2-(Pentafluorophenyl)-6,7-dihydro-5*H*-pyrrolo[2,1-*c*][1,2,4]triazol-2-ium tetrafluoroborate (**S3**) was synthesized according to our previous report.[2] Unless otherwise noted, chemicals obtained from commercial suppliers were used without further purification.

All the photophysical properties (*i.e.*, UV-vis absorption, photoluminescence (PL), and excitation spectra, PL quantum yields (PLQYs), and PL lifetimes) were evaluated at room temperature (RT) in air unless otherwise noted. Spectral-grade cyclohexane (Wako Chemical) and KF-96-3000CS (Shin-Etsu Chemical) were used in the spectroscopic measurement of the solutions. UV-vis absorption spectra in solution were acquired using a Shimadzu UV-3150 or JASCO V-750 spectrometer or a JASCO FP-8200 spectrofluorometer. Diffuse reflectance spectra of the solids were acquired using a SHIMADZU UV-3150 spectrometer and a SHIMADZU ISR-3100 integrating sphere attachment. Crystalline solids were embedded in a $BaSO_4$ plate. Kubelka-Munk conversion of the obtained

spectra gave solid-state absorption spectra. UV-vis absorption spectra of solvent-free liquids were acquired using a JASCO V-750 spectrometer or a SHIMADZU UV-3150 spectrometer and a SHIMADZU ISR-3100 integrating sphere attachment. The thin liquid film was prepared by either drop-casting or spin-coating the $CHCl_3$ solution onto a quartz plate, and then dried under vacuum to remove solvents before the measurements. PL spectra of the solutions, crystals, and the liquid were acquired using a JASCO FP-8200 spectrofluorometer. Φ_p value of **1DMOS-BrTn** in solution was determined by the relative method using quinine sulfate as a standard.[3] Φ_p values of other samples were determined as an average of four measurements by the absolute method using a Hamamatsu photonics C9920-02 spectrometer with an integrating sphere. PL lifetime measurements were performed using a HORIBA DeltaFlex multichannel scaling system using DeltaDiode for excitation (368 nm).

2. Synthesis Procedures

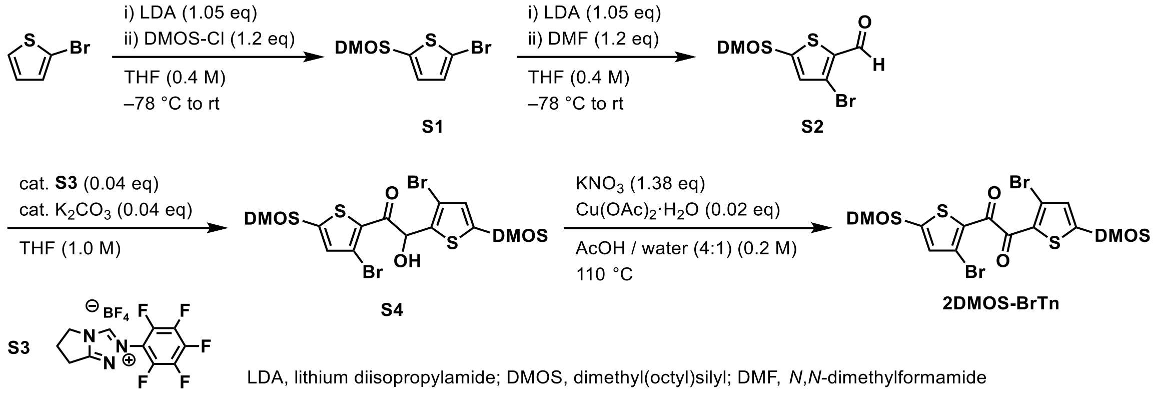

Scheme S1 Synthesis of **2DMOS-BrTn**.

2-bromo-5-[dimethyl(octyl)silyl]thiophene (S1)

To a 50 mL 2-neck flask were added diisopropylamine (2.5 mL, 17.7 mmol) and anhydrous THF (10 mL). The flask was cooled to –30 °C, and BuLi (2.69 M in hexane, 6.2 mL, 16.7 mmol) was added dropwise. The mixture was stirred for 20 min at 0 °C to prepare LDA solution. To another 200 mL 2-neck flask were added 2-bromothiophene (1.55 mL, 16.0 mmol) and THF (30 mL). The flask was cooled to –78 °C and the freshly prepared LDA solution (cooled to –78 °C) was added dropwise via cannula. The mixture was stirred for 30 min at the same temperature. To the mixture was added chlorodimethyl(octyl)silane (4.5 mL, 19.0 mmol) dropwise. The mixture was stirred for 1 h at –78 °C before the cooling bath was removed, and the mixture was stirred overnight at RT. The reaction was quenched by adding aq. NH_4Cl and Et_2O. The organic layer was separated, and the aqueous layer was extracted with Et_2O. The combined organic extracts were washed with brine, dried over $MgSO_4$, filtered, and then evaporated. The residue was purified by silica-gel column chromatography (eluent: hexane) and recycling gel-permeation chromatography to give **S1** (4.87 g, 91%) as an orange oil. **^{1}H NMR** (400 MHz, $CDCl_3$) δ: 7.07 (d, *J* = 3.7 Hz, 1H), 6.97 (d, *J* = 3.7 Hz, 1H), 1.35–1.23 (m, 12H), 0.88 (t, *J* = 6.9 Hz, 3H), 0.75–0.71 (m, 2H), 0.27 (s, 6H). **^{13}C NMR** (100 MHz, $CDCl_3$) δ: 142.46, 134.52, 131.08, 116.60, 33.42, 31.90, 29.22, 23.66, 22.66, 16.37, 14.10, –2.04. **APCI-HRMS** (*m*/*z*): $[M+H]^+$ calcd for $C_{14}H_{25}BrSSi$, 335.0682; found, 335.0684.

3-bromo-5-[dimethyl(octyl)silyl]thiophene-2-carbaldehyde (S2)

To a 200 mL 2-neck flask were added **S1** (4.80 g, 14.4 mmol) and THF (28 mL). LDA was prepared by the same method described above from diisopropylamine (2.2 mL, 15.6 mmol), BuLi (2.69 M in hexane, 5.6 mL, 15.1 mmol), and THF (8 mL). To the reaction

mixture containing thiophene was transferred LDA dropwise via cannula at –78 °C. The resulting mixture was stirred for 2 h, and then DMF (1.3 mL, 16.8 mmol) was added dropwise. After 15min, the cooling bath was removed, and the mixture was stirred overnight at RT. The reaction was quenched by adding aq. NH_4Cl and Et_2O. The organic layer was separated, and the aqueous layer was extracted with Et_2O. The combined organic extracts were washed with brine, dried over $MgSO_4$, filtered, and then evaporated. The residue was purified by silica-gel column chromatography (eluent: hexane/CH_2Cl_2 4:1 to 2:1) to give **S2** (3.44 g, 66%) as an orange oil. **^{1}H NMR** (400 MHz, $CDCl_3$) δ: 9.96 (s, 1H), 7.20 (s, 1H), 1.34-1.23 (m, 12H), 0.88, (t, J = 6.9 Hz, 3H), 0.80–0.76 (m, 2H), 0.33 (s, 6H). **^{13}C NMR** (100 MHz, $CDCl_3$) δ: 182.58, 151.74, 140.61, 137.72, 120.67, 33.29, 31.85, 29.17, 29.14, 23.52, 22.62, 15.84, 14.07, –2.45. **ESI-HRMS** (*m*/*z*): $[M+H]^+$ calcd for $C_{15}H_{26}BrOSSi$, 361.0652; found, 361.0648.

1,2-bis(3-bromo-5-(dimethyl(octyl)silyl)thiophen-2-yl)-2-hydroxyethan-1-one (S4)

To a Schlenk tube were added K_2CO_3 (34.3 mg, 0.248 mmol) and triazolium salt **S3** (90.0 mg, 0.248 mmol), and the tube was shortly evacuated and backfilled with N_2 three times. THF (3 mL) was added, and the suspension was stirred for 15 minutes. To the pale red suspension was transferred a solution of **S2** (2.24 g, 6.20 mmol) in THF (1 mL) and washed with additional THF (2 mL). The mixture was stirred for 48 h at RT, and then quenched by adding aq. NH_4Cl and CH_2Cl_2. The organic layer was separated, and the aqueous layer was extracted with CH_2Cl_2. The combined organic extracts were dried over $MgSO_4$, filtered, and then evaporated. The residue was purified by silica-gel column chromatography (eluent: hexane/CH_2Cl_2 4:1 to 2:1) to give **S4** (1.25 g, 56%) as an orange liquid. **^{1}H NMR** (400 MHz, $CDCl_3$) δ: 7.15 (s, 1H), 7.05 (s, 1H), 6.12 (d, J = 6.2 Hz, 1H), 4.37 (d, J = 6.2 Hz, 1H), 1.30–1.22 (m, 24H), 0.89–0.86 (m, 6H), 0.76–0.68 (m, 4H), 0.29 (s, 6H), 0.24 (s, 6H). **^{13}C NMR** (100 MHz, $CDCl_3$) δ: 188.85, 150.65, 142.52, 139.74, 139.38, 136.84, 135.79, 118.77, 114.22, 71.48, 33.33, 33.30, 31.87, 29.18, 29.16, 23.54, 23.51, 22.63, 16.05, 15.80, 14.09, –2.27, –2.42, –2.46. **ESI-HRMS** (*m*/*z*): $[M+Na]^+$ calcd for $C_{30}H_{50}Br_2O_2S_2Si_2Na$, 745.1035; found, 745.1027.

1,2-bis(3-bromo-5-(dimethyl(octyl)silyl)thiophen-2-yl)ethane-1,2-dione (2DMOS-BrTn)

To a Schlenk tube were added **S4** (1.25 g, 1.72 mmol), KNO_3 (240 mg, 2.37 mmol), $Cu(OAc)_2 \cdot H_2O$ (6.9 mg, 35 μmol), and AcOH/H_2O (4:1, 9 mL) in air. The mixture was stirred overnight at 110 °C. At RT, the mixture was quenched by adding H_2O and CH_2Cl_2. The organic layer was separated, and the aqueous layer was extracted with CH_2Cl_2 three

times. The residue was purified by recycling gel-permeation chromatography and silica-gel column chromatography (eluent: hexane/AcOEt 30:1) to give **2DMOS-BrTn** (0.866 g, 70%) as an orange liquid. **^{1}H NMR** (400 MHz, $CDCl_3$) δ: 7.24 (s, 1H), 1.34–1.24 (m, 12H), 0.88 (t, J = 6.9 Hz, 3H), 0.82–0.78 (m, 2H), 0.35 (s, 6H). **^{13}C NMR** (100 MHz, $CDCl_3$) δ: 182.25, 153.76, 139.05, 135.69, 120.06, 33.30, 31.86, 29.17, 29.14, 23.52, 22.63, 15.86, 14.09, –2.42. **EA** Calcd for $C_{30}H_{48}Br_2O_2S_2Si_2$: C, 49.99; H, 6.71. Found: C, 49.93; H, 6.65.

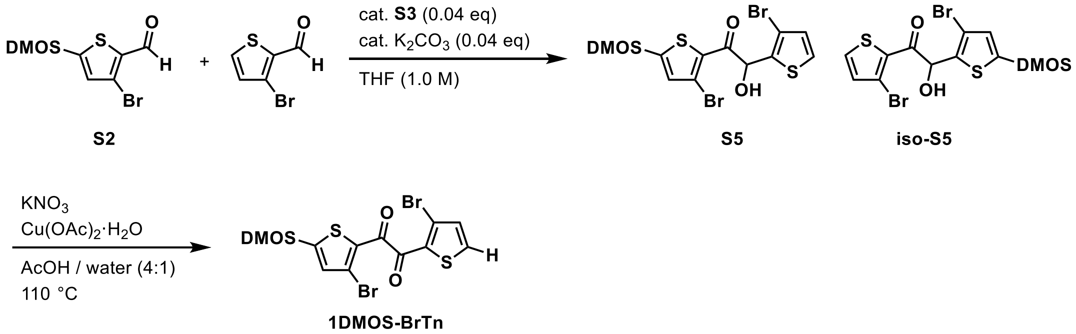

Scheme S2 Synthesis of **1DMOS-BrTn**.

1-(3-bromo-5-(dimethyl(octyl)silyl)thiophen-2-yl)-2-(3-bromothiophen-2-yl)ethane-1,2-dione (1DMOS-BrTn)

To a Schlenk tube were added K_2CO_3 (38 mg, 0.28 mmol) and triazolium salt **S3** (100 mg, 0.27 mmol), and the tube was shortly evacuated and backfilled with Ar three times. THF (3 mL) was added via a syringe, and the suspension stirred for 15 minutes. To the pale red suspension was added a solution of 3-bromothiophen-2-carbaldehyde (1.31 g, 6.85 mmol, 1.0 equiv.) and **S2** (2.35 g, 6.50 mmol, 0.95 equiv.) in THF (1 mL) and washed with additional THF (2 mL). The mixture was stirred for 48 h at RT, and then quenched by adding aq. NH_4Cl and CH_2Cl_2. The organic layer was separated, and the aqueous layer was extracted with CH_2Cl_2. The combined organic extracts were dried over $MgSO_4$, filtered, and then evaporated. The residue was purified by silica-gel column chromatography (eluent: hexane/CH_2Cl_2 1:1) to collect the fraction containing benzoins **S5** and **iso-S5** as an orange liquid. To a Schlenk tube were added the mixture (1.12 g), KNO_3 (279 mg, 2.76 mmol), $Cu(OAc)_2$·H_2O (8.0 mg, 40 μmol), and AcOH/H_2O (4:1, 10 mL). The mixture was stirred overnight at 110 °C. After cooling to RT, the mixture was quenched by adding H_2O and CH_2Cl_2. The organic layer was separated, and the aqueous layer was extracted with CH_2Cl_2 three times. The residue was purified by silica-gel col-

umn chromatography (eluent: hexane/CH_2Cl_2 = 2:1) and recycling gel permeation chromatography to give **1DMOS-BrTn** (0.548 g, 15% for two steps) as an orange liquid. **^{1}H NMR** (400 MHz, $CDCl_3$) δ: 7.75 (d, *J* = 5.3 Hz, 1H), 7.24, (s, 1H), 7.19, (d, *J* = 5.3 Hz, 1H), 1.34–1.24 (m, 12H), 0.88 (t, *J* = 6.9 Hz, 3H), 0.82–0.79 (m, 2H), 0.35 (s, 6H). **^{13}C NMR** (100 MHz, $CDCl_3$) δ: 182.67, 182.15, 153.99, 139.07, 136.38, 135.77, 133.28, 132.10, 120.09, 119.69, 33.30, 31.86, 29.17, 29.14, 23.52, 22.63, 15.85, 14.09, –2.42. **EA** Calcd for $C_{20}H_{26}Br_2O_2S_2Si$: C, 43.64; H, 4.76. Found: C, 43.49; H, 4.76.

3. Additional Photophysical Data

Steady-state PL spectra in air and under Ar

Photophysical properties in cyclohexane solution were carefully evaluated following the protocol established previously.[4] Briefly, the PL quantum yields under Ar were evaluated before and after acquisition of decay curves to ensure the same degree of deoxygenation in determining $\Phi_{\rm p}$ and $\tau_{\rm p}$.

To evaluate the effect of oxygen on the photophysical properties of the solvent-free liquids, liquid film was prepared onto an inside wall of a quartz cell (path length: 10 mm) with extended tube at the top (ϕ = 8 mm, l = 55 mm). The cell was placed in a vacuum chamber and dried *in vacuo* overnight before the measurements.

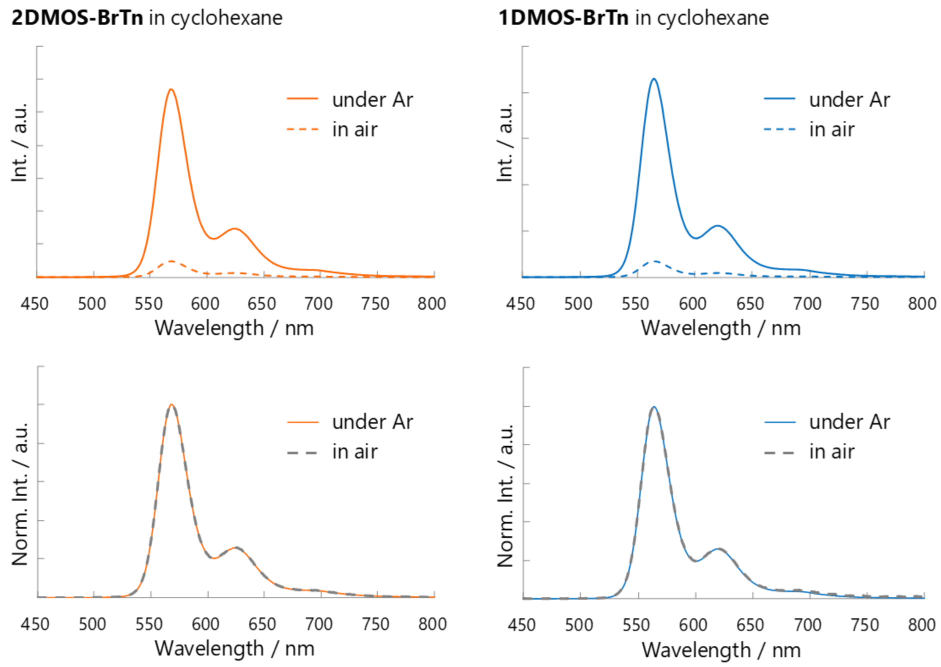

Figure S1. Steady-state PL spectra of **2DMOS-BrTn** (left) and **1DMOS-BrTn** (right) in cyclohexane (1.0 × 10^{-5} M) under Ar and in air. Excitation wavelength was 368 nm. The emission was enhanced under Ar without changing the spectral shape.

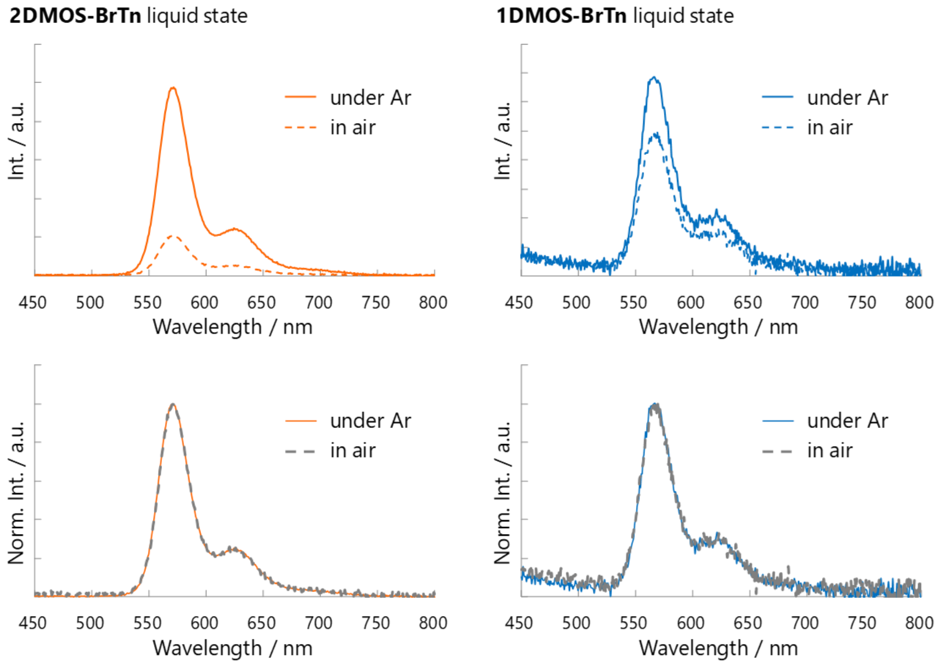

Figure S2. Steady-state PL spectra of liquid state **2DMOS-BrTn** (left) and **1DMOS-BrTn** (right) under Ar and in air. Excitation wavelength was 368 nm. The emission was enhanced under Ar without changing the spectral shape.

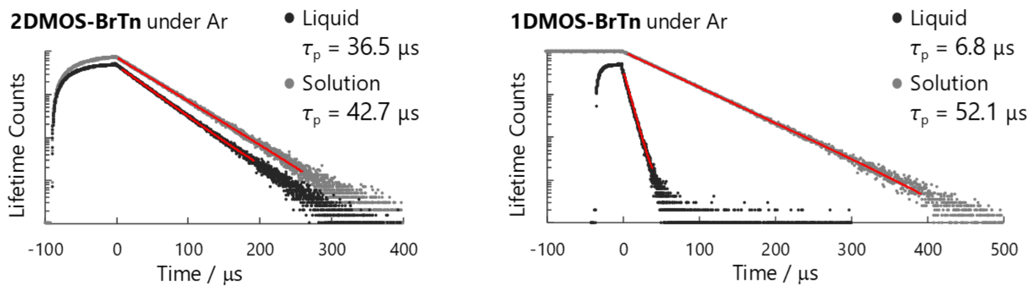

Figure S3. PL decay curves of **2DMOS-BrTn** (left) and **1DMOS-BrTn** (right) in solution (1.0×10^{-5} M in cyclohexane) and solvent-free liquid state acquired under Ar. Red lines denote the fit to the curve. Excitation wavelength was 368 nm.

Temperature dependence on the steady-state PL spectra

The film of solvent-free liquid **2DMOS-BrTn** was prepared inside the wall of a quartz tube (diameter of 5 mm). The PL spectra were acquired over a temperature range from –50 to 40 °C in 10 °C steps using a JASCO FP-8200 spectrofluorometer equipped with a UNISOK CoolSpeK USP-203-B cryostat (λ_{ex} = 320 nm). The intensity increased at lower

temperatures in a good Arrhenius-type relationship, with a slight sharpening of the spectral shape without the emergence of new peaks, indicating that the emission is phosphorescence-dominated rather than thermally-activated delayed fluorescence.

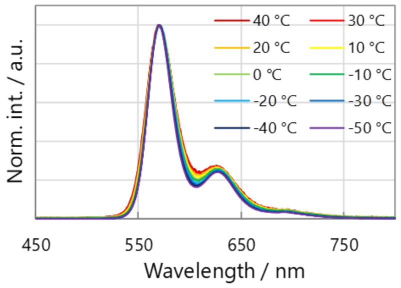

Figure S4. Temperature dependence of normalized steady-state PL spectra of solvent-free liquid **2DMOS-BrTn** in air, excited at 320 nm.

Transient absorption spectroscopy

The femtosecond transient absorption (TA) measurement was performed by a home-built setup utilizing the pump-probe method. A Yb:KGW regenerative amplifier system (Light Conversion, PHAROS, pulse duration: 190 fs, repetition rate: 1 kHz, pulse energy: 1 mJ/pulse, wavelength: 1030 nm) was employed as the light source. The amplifier output was divided into two pulses for generation of the pump and probe pulses. The sample was pumped with the second harmonic generation at 320 nm from the output of an optical parametric amplifier system (Light Conversion, ORPHEUS) tuned at 640 nm. The fluence of pump pulse was 0.47 mJ/cm^2 at the sample position. To generate the broadband probe pulse (500–950 nm), a 3 mm-thick sapphire crystal was employed. The polarization angle between the pump and probe pulse was adjusted to the magic-angle configuration (54.7°). The probe pulse that passed through the sample was dispersed using a polychromator (JASCO, CT-10, 300 grooves/500 nm), and the resulting spectra were recorded by a multichannel detection system equipped with a CMOS sensor (UNISOKU, USP-PSMM-NP).

UV-vis absorption and excitation spectra of the liquids

Although the spectral shape was broadened in the liquid state, it is due to the breakdown of the Beer–Lambert law, as a sufficiently thin film (prepared by spin-coating method) provided a spectrum that almost overlapped with that in solution (Fig. 4e).

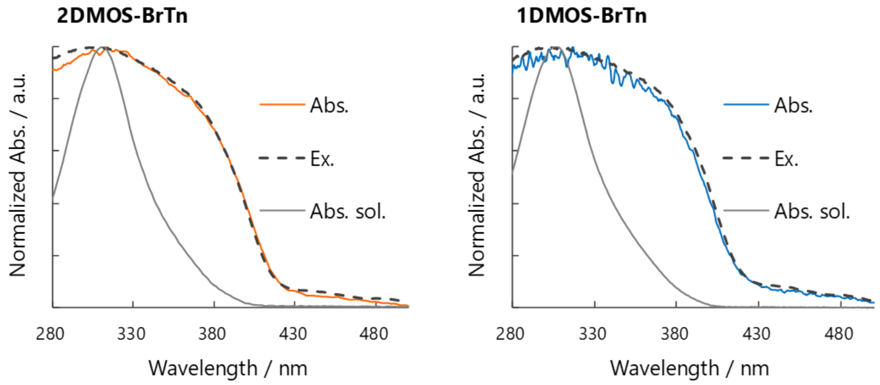

Figure S5. UV-vis absorption (solid line) and excitation spectra (broken line) of liquid **2DMOS-BrTn** (left) and **1DMOS-BrTn** (right) at RT in air. Emission wavelength was 570 nm. Corresponding UV-vis absorption spectra in solution (1.0×10^{-5} M in cyclohexane) were shown in grey line.

Photophysical properties in silicone oil

The photophysical properties in silicone oil KF-96-3,000CS ($\eta = 2.9$ Pa.s) were evaluated for the solution with absorbance at 368 nm of 0.02 and 0.03 for **2DMOS-BrTn** and **1DMOS-BrTn**, respectively. Φ_p (τ_p) were determined to be 2.0% (4.0 μs) and 1.8% (3.8 μs) for **2DMOS-BrTn** and **1DMOS-BrTn**, respectively. These values were similar to those in cyclohexane ($\eta = 0.93$ mPa.s at 22 °C). In addition, the absorption and emission maxima in silicone oil were almost same as those in cyclohexane. These results suggest the negligible effect of viscosity.

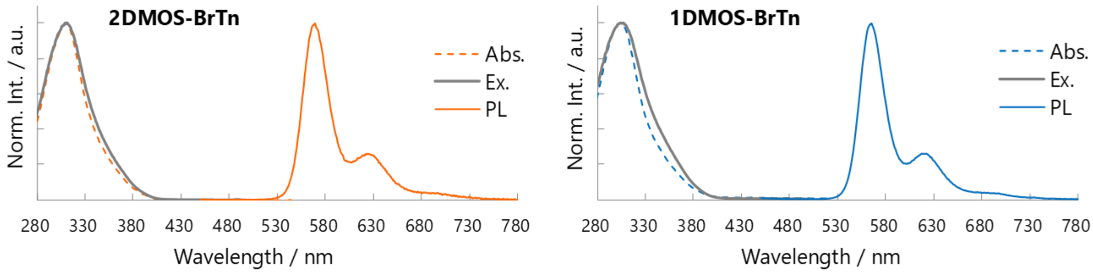

Figure S6. UV-vis absorption (broken orange/blue line), PL (solid orange/blue line), and excitation spectra (solid grey line) of **2DMOS-BrTn** (left) and **1DMOS-BrTn** (right) in silicone oil KF-96-3,000CS at RT in air. Excitation wavelength was 368 nm and emission wavelength was 570 nm. PL spectra were acquired with U340 bandpass filter (HOYA).

4. NMR Spectra

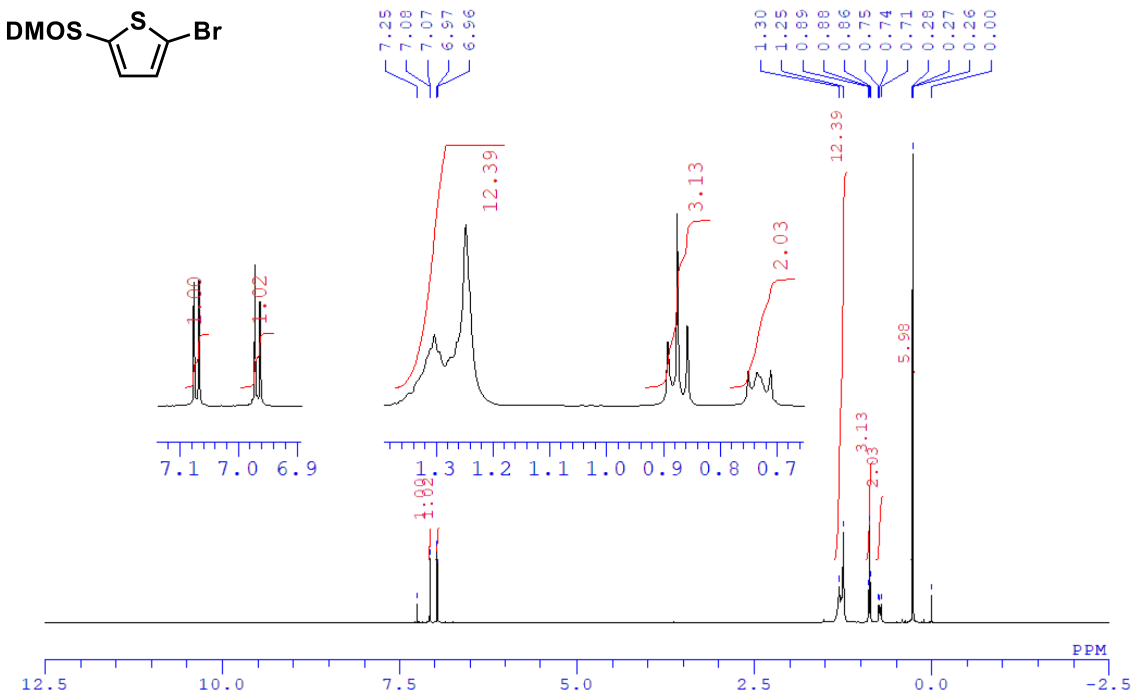

Figure S7. ^{1}H NMR spectrum of **S1** ($CDCl_3$, 400 MHz).

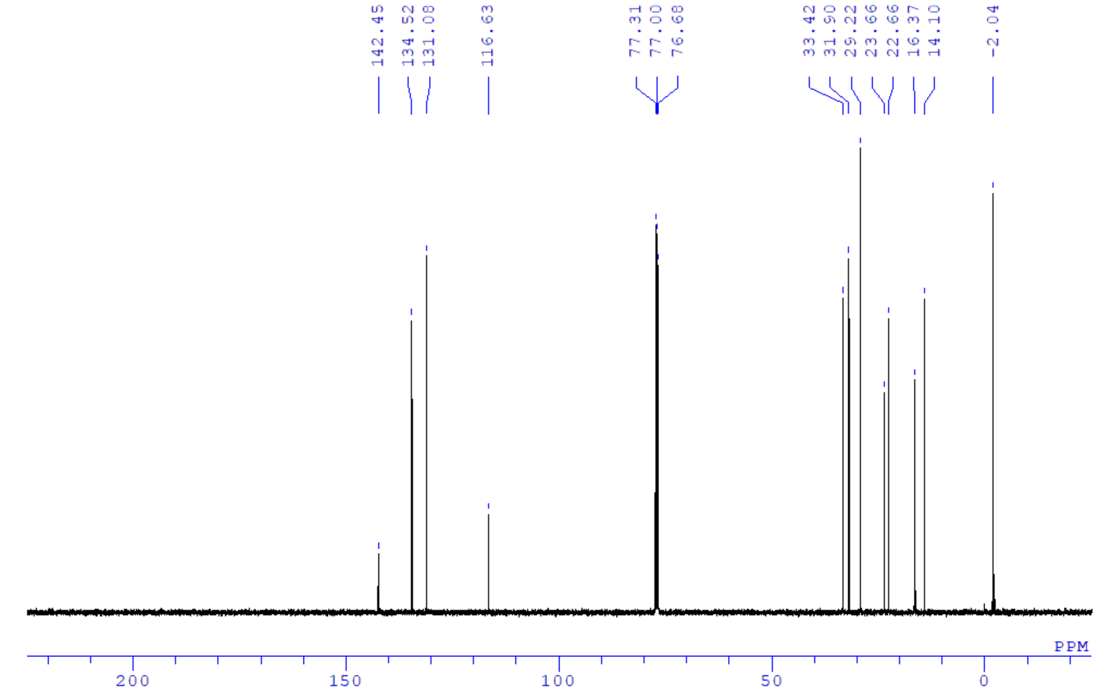

Figure S8. ^{13}C NMR spectrum of **S1** ($CDCl_3$, 100 MHz).

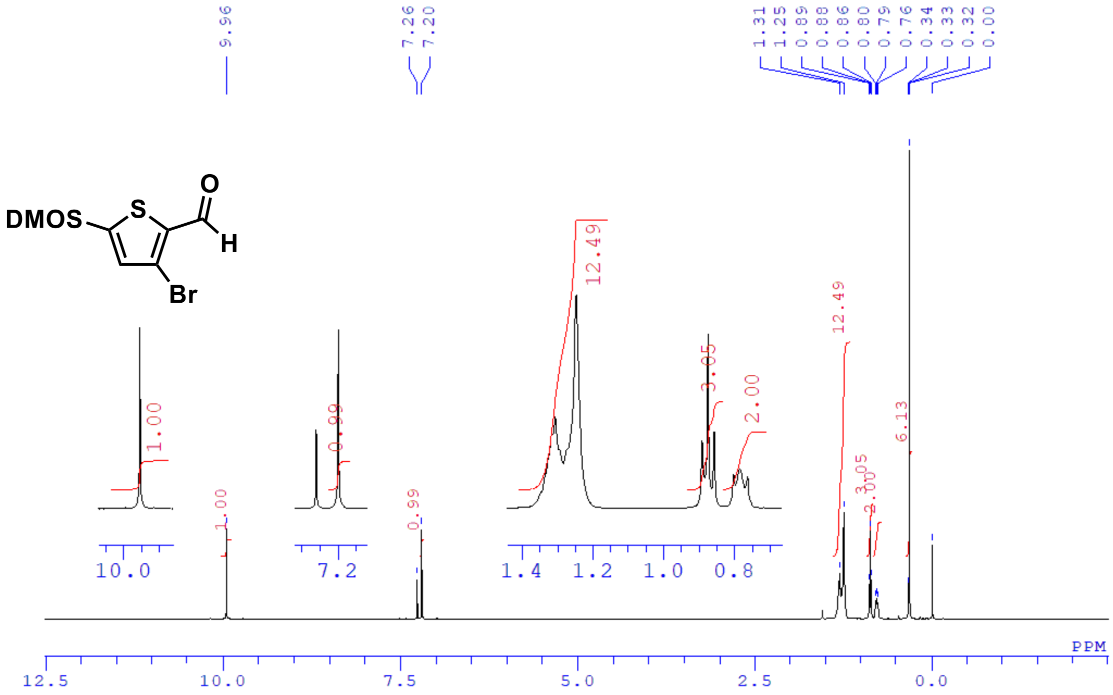

Figure S9. ^{1}H NMR spectrum of **S2** ($CDCl_3$, 400 MHz).

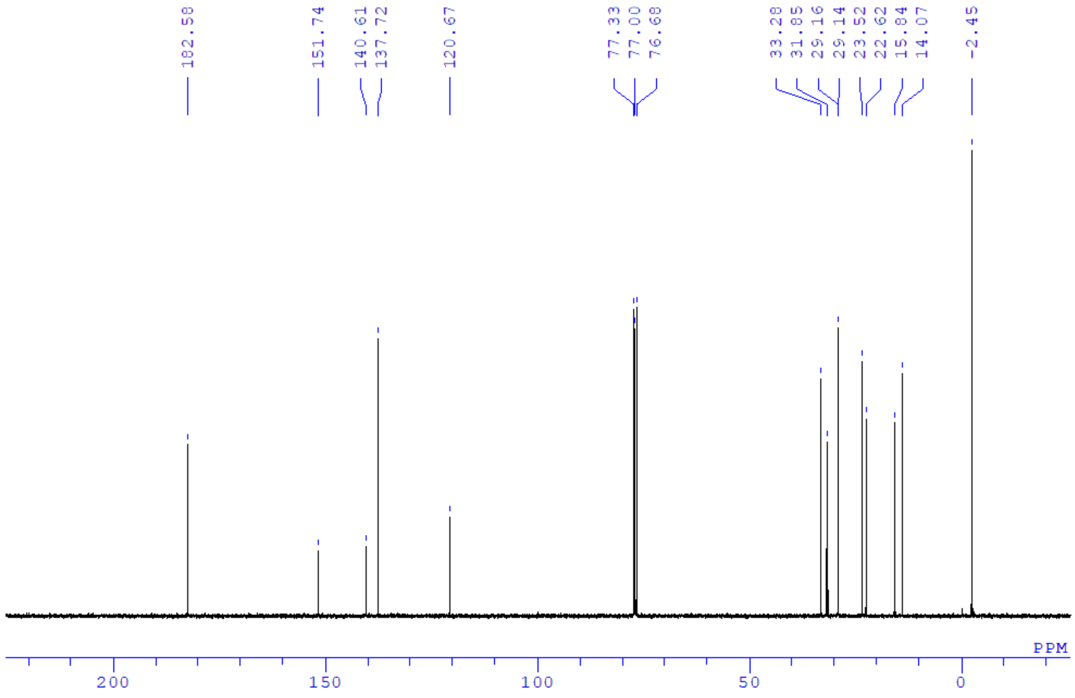

Figure S10. ^{13}C NMR spectrum of **S2** ($CDCl_3$, 100 MHz).

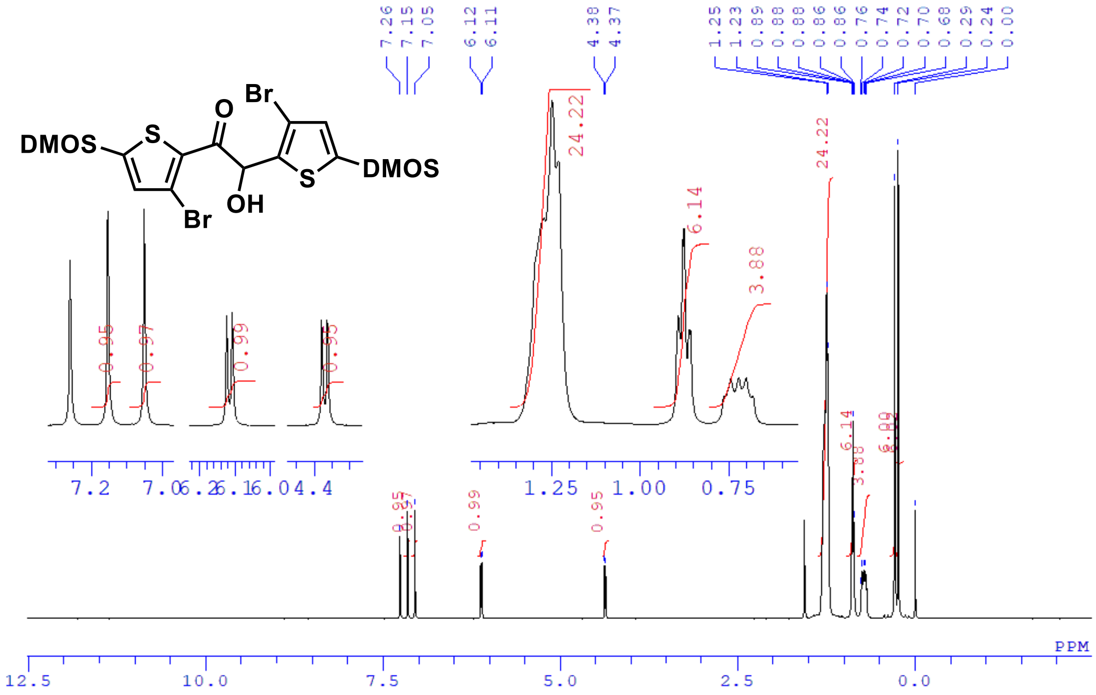

Figure S11. ^{1}H NMR spectrum of **S4** ($CDCl_3$, 400 MHz).

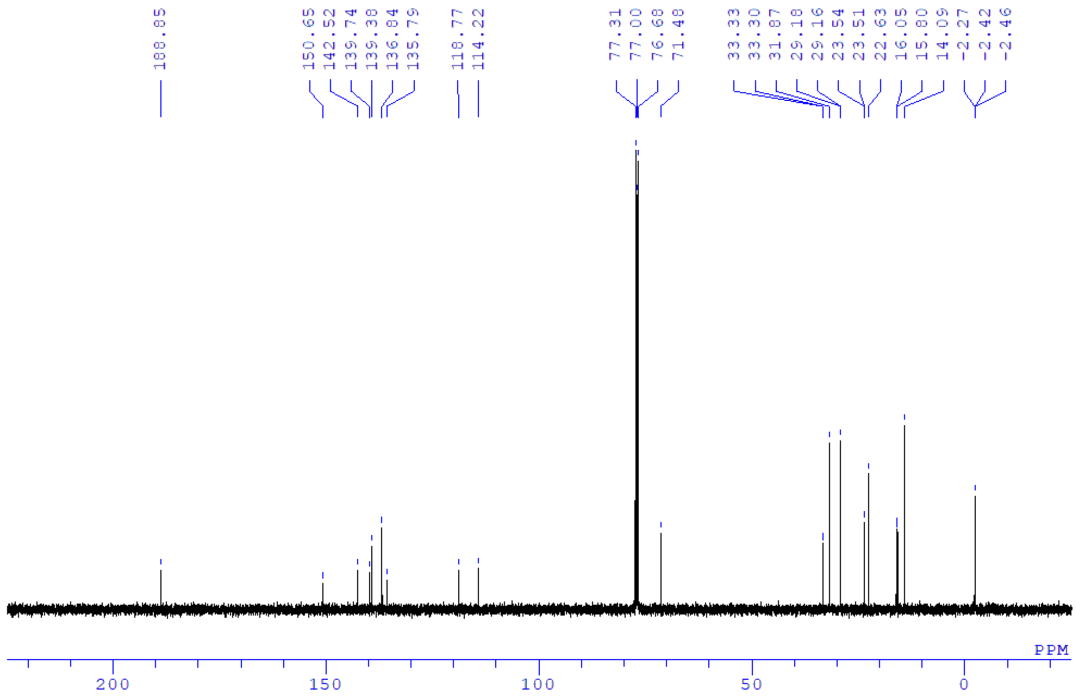

Figure S12. ^{13}C NMR spectrum of **S4** ($CDCl_3$, 100 MHz).

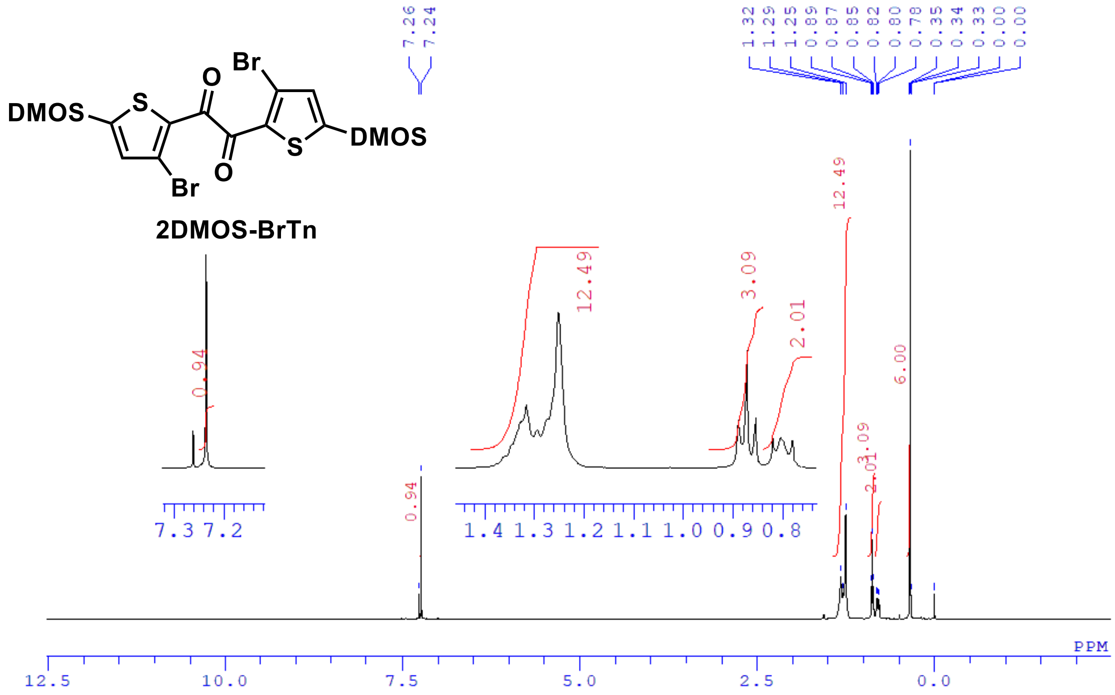

Figure S13. ^{1}H NMR spectrum of **2DMOS-BrTn** ($CDCl_3$, 400 MHz).

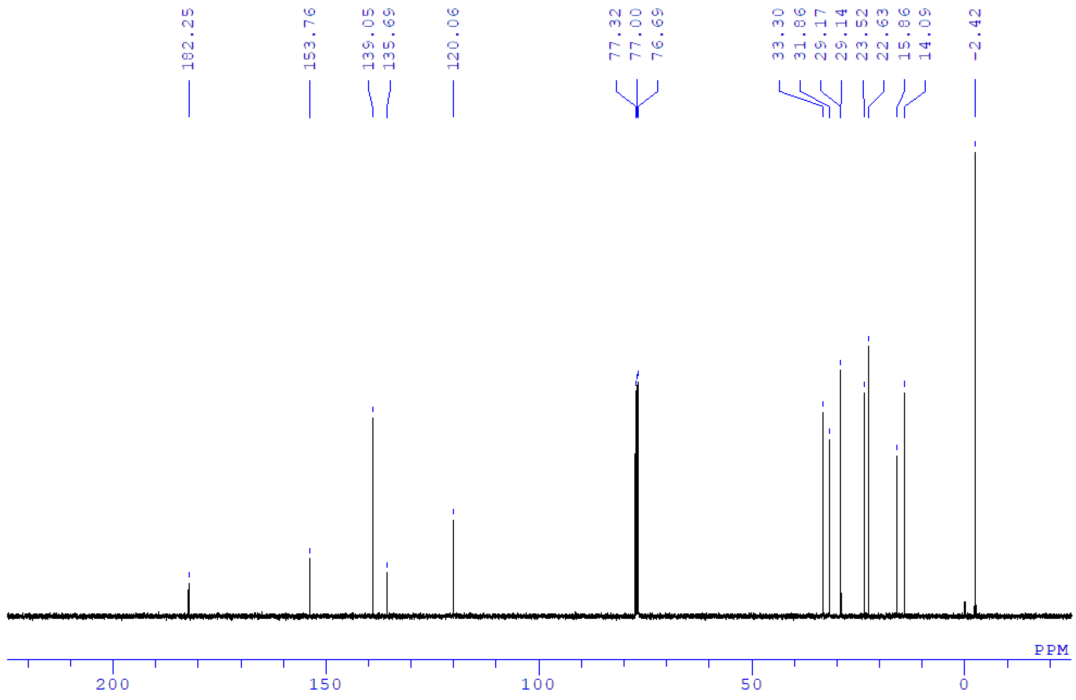

Figure S14. ^{13}C NMR spectrum of **2DMOS-BrTn** ($CDCl_3$, 100 MHz).

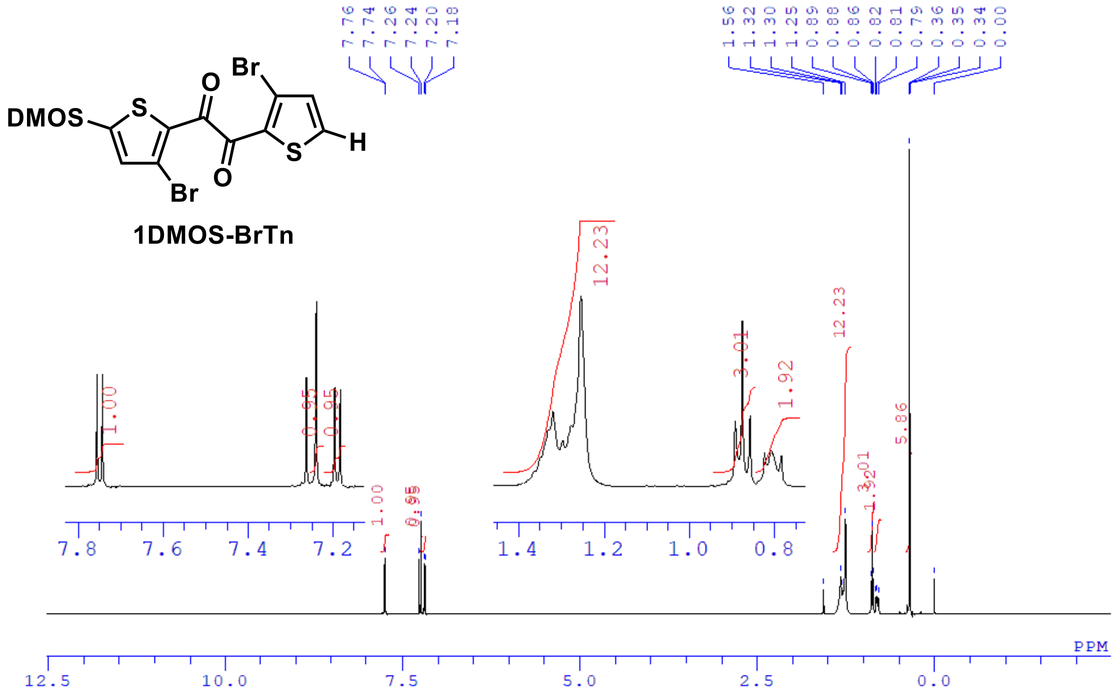

Figure S15. ^{1}H NMR spectrum of **1DMOS-BrTn** ($CDCl_3$, 400 MHz).

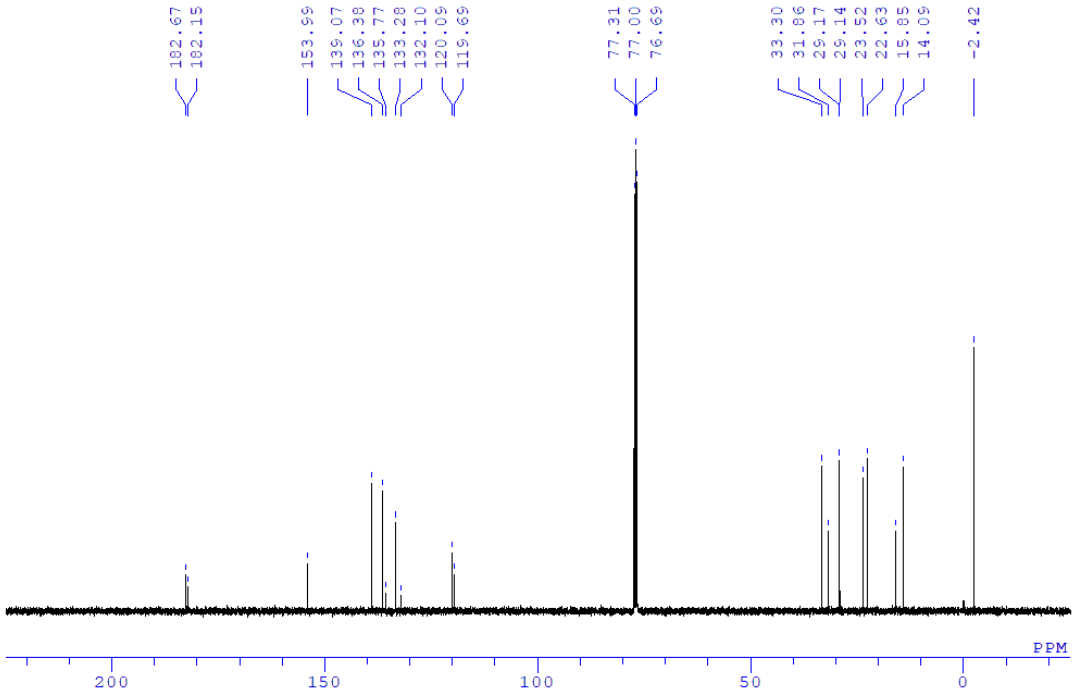

Figure S16. ^{13}C NMR spectrum of **1DMOS-BrTn** ($CDCl_3$, 100 MHz).

5. References

1. A. B. Pangborn, M. A. Giardello, R. H. Grubbs, R. K. Rosen and F. J. Timmers, *Organometallics*, 1996, **15**, 1518-1520.
2. Y. Tani, M. Terasaki, M. Komura and T. Ogawa, *J. Mater. Chem. C*, 2019, **7**, 11926-11931.
3. (a) K. Suzuki, A. Kobayashi, S. Kaneko, K. Takehira, T. Yoshihara, H. Ishida, Y. Shiina, S. Oishi and S. Tobita, *Phys. Chem. Chem. Phys.*, 2009, **11**, 9850–9860; (b) A. M. Brouwer, *Pure Appl. Chem.*, 2011, **83**, 2213-2228.
4. Y. Tani, K. Miyata, E. Ou, Y. Oshima, M. Komura, M. Terasaki, S. Kimura, T. Ehara, K. Kubo, K. Onda and T. Ogawa, *Chem. Sci.*, 2024, **15**, 10784-10793.
